# Supplementary figures and images for: Clustering long-term health conditions among 67728 people with multimorbidity using electronic health records in Scotland
Source: PLoS One. 2023 Nov 29;18(11):e0294666. doi: 10.1371/journal.pone.0294666 (PMC10686427; doi:10.1371/journal.pone.0294666)

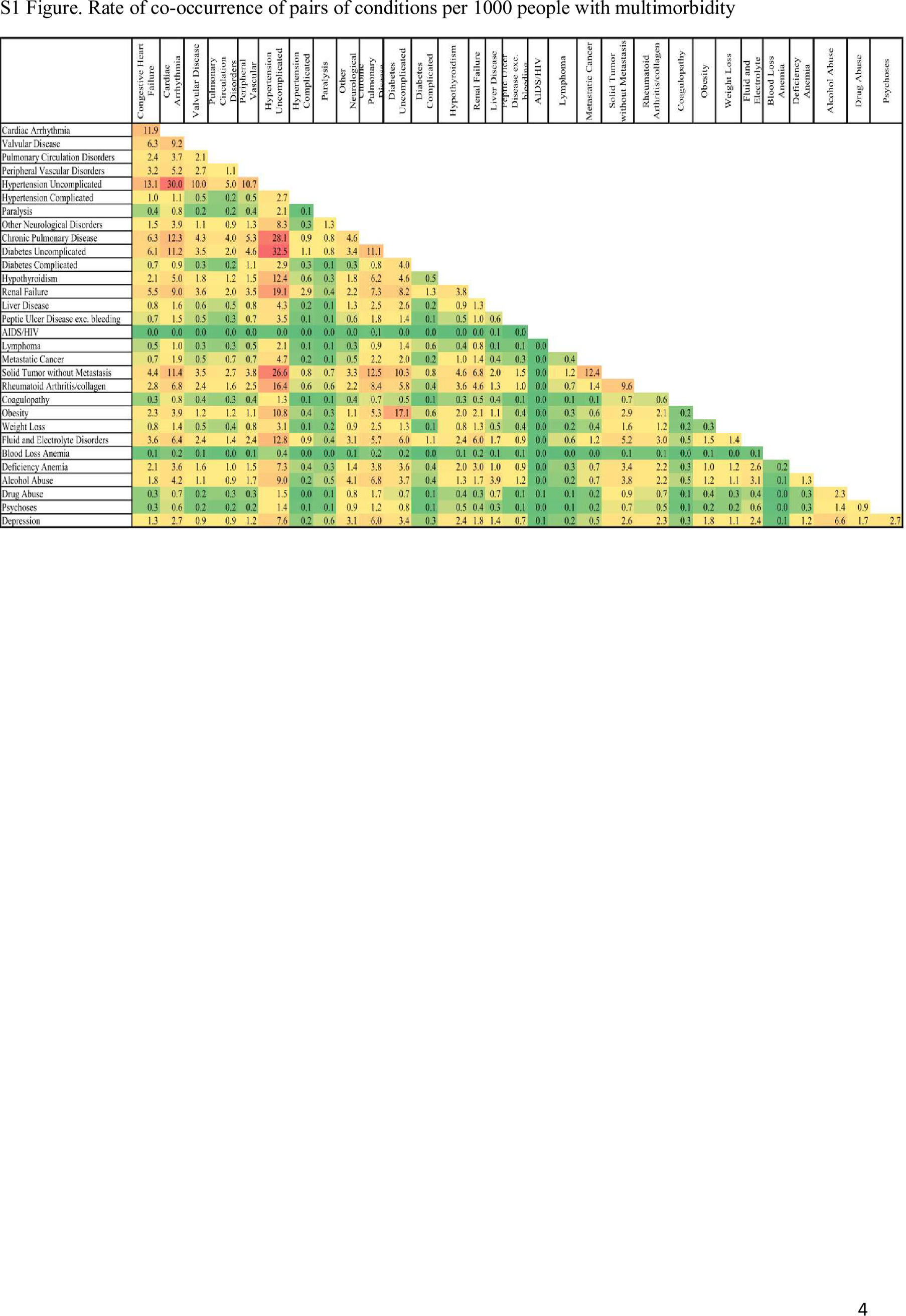

Supplement: S1 Fig — (TIF) [file pone.0294666.s001.tif]

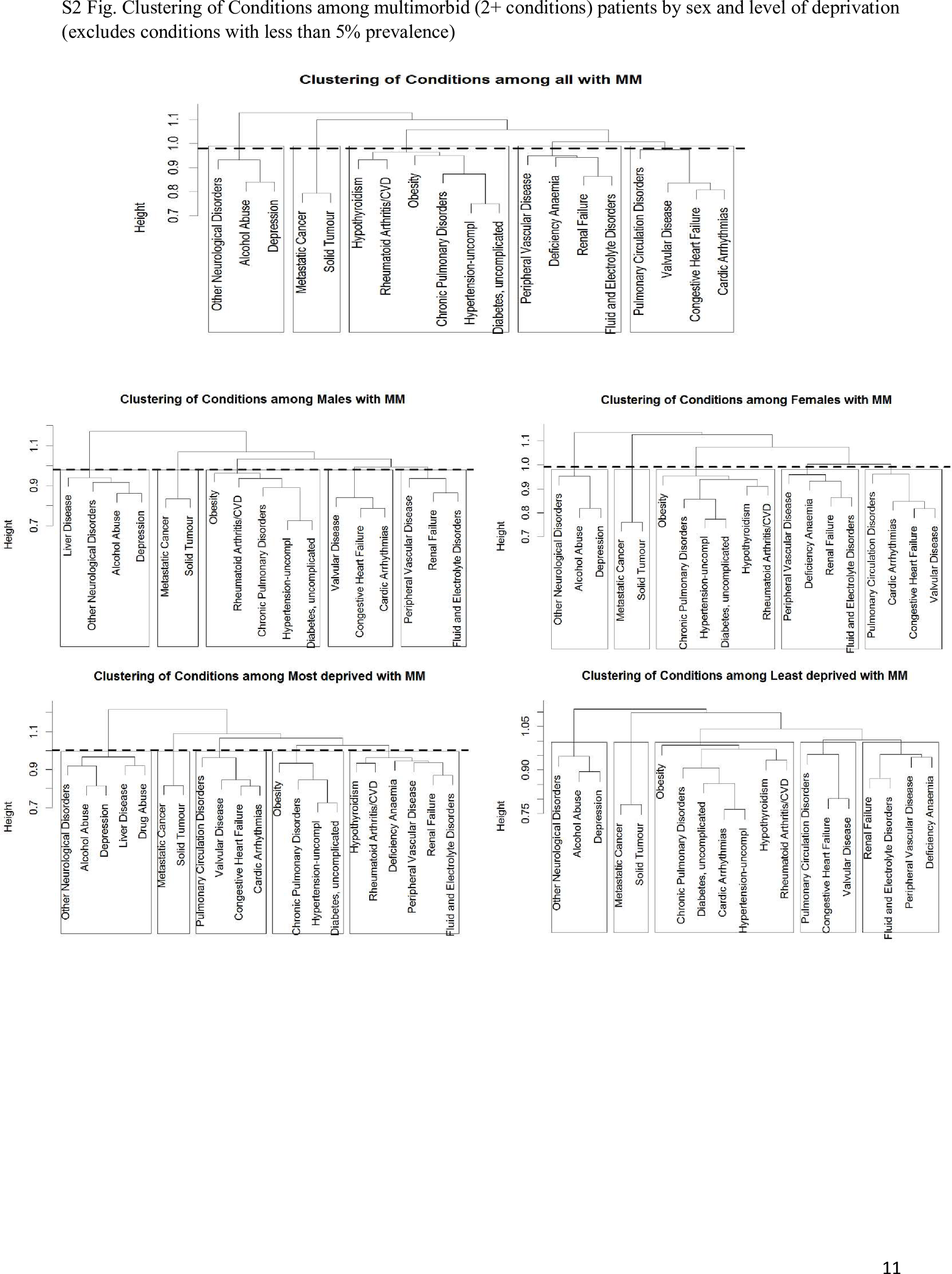

Supplement: S2 Fig — (TIF) [file pone.0294666.s002.tif]

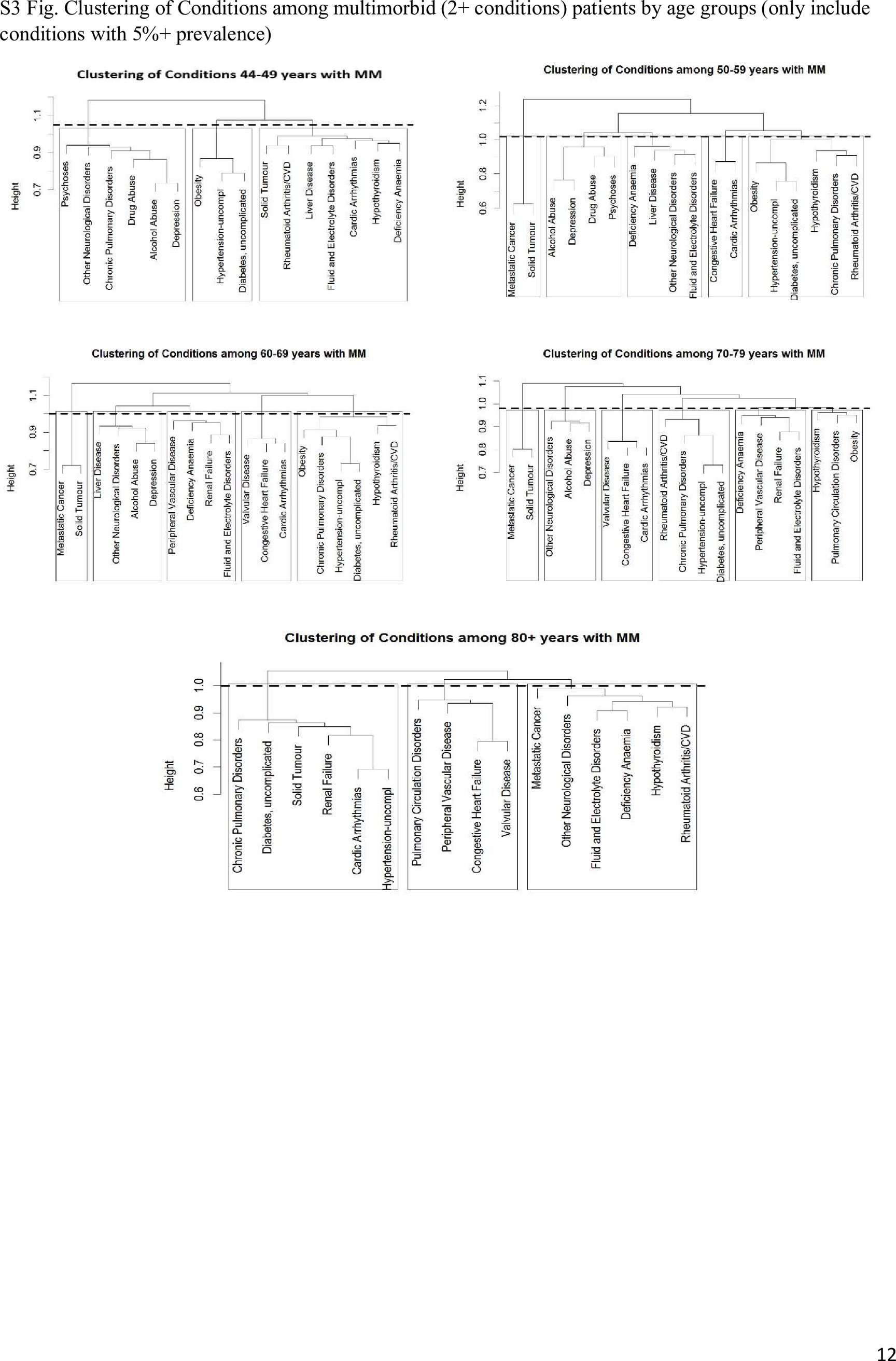

Supplement: S3 Fig — (TIF) [file pone.0294666.s003.tif]

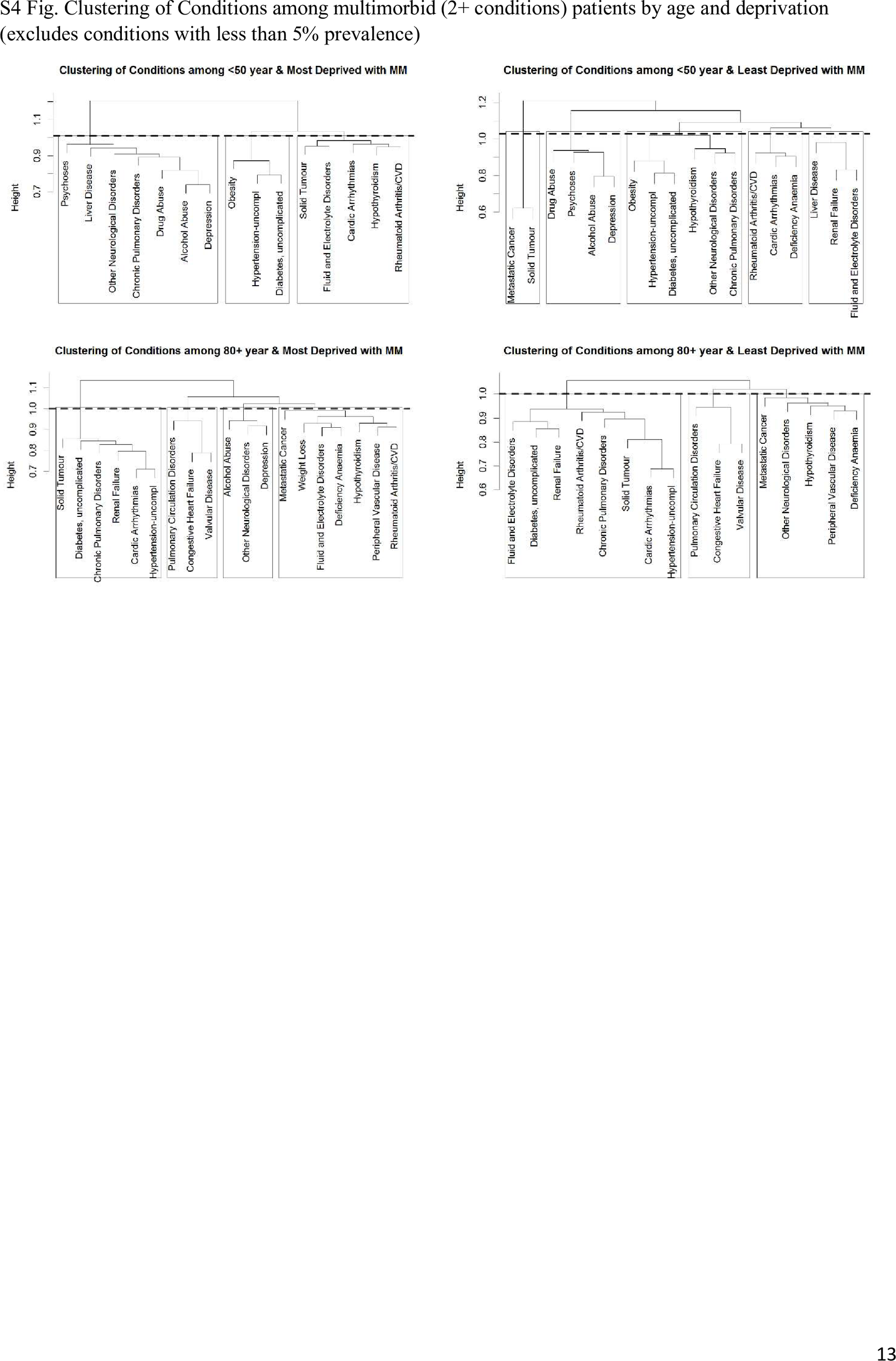

Supplement: S4 Fig — (TIF) [file pone.0294666.s004.tif]
