# Supplementary material for: Clustering long-term health conditions among 67728 people with multimorbidity using electronic health records in Scotland
Source: PLoS One. 2023 Nov 29;18(11):e0294666. doi: 10.1371/journal.pone.0294666 (PMC10686427; doi:10.1371/journal.pone.0294666)
Supplement: S1 Table — (PDF) [file pone.0294666.s005.pdf]

Supporting Information

S1 Table. List of Elixhauser Index conditions, abbreviations, ICD10 codes

Characteristics	Abbreviations	ICD codes
AIDS/HIV	AIDS	B20,B21,B22,B24
Alcohol Abuse	Alcohol	F10,E52,T51,G62.1,I42.6,K29.2,K70.0,K70.3,K70.9,Z50.2,Z71.4,Z72.1
Blood Loss Anemia	Blane	D50.0
Cardiac Arrhythmia	Carit	I47,I48,I49,I44.1,I44.2,I44.3,I45.6,I45.9,R00.0,R00.1,R00.8,T82.1,Z45.0, Z95.0
Chronic Pulmonary Disease	CPD	J40,J41,J42,J43,J44,J45,J46,J47,J60,J61,J62,J63,J64,J65,J66,J67,I27.8,I27.9, J68.4,J70.1,J70.3
Coagulopathy	Coag	D65,D66,D67,D68,D69.1,D69.3,D69.4,D69.5,D69.6
Congestive Heart Failure	CHF	I43,I50,I09.9,I11.0,I13.0,I13.2,I25.5,I42.0,I42.5,I42.6,I42.7,I42.8,I42.9,P29.0
Deficiency Anemia	Dane	D51,D52,D53,D50.8,D50.9
Depression	Depre	F32,F33,F20.4,F31.3,F31.4,F31.5,F34.1,F41.2,F43.2
Diabetes with Chronic Complication	Diabc	E10.2,E10.3,E10.4,E10.5,E10.6,E10.7,E10.8,E11.2,E11.3,E11.4,E11.5,E11.6, E11.7,E11.8,E12.2,E12.3,E12.4,E12.5,E12.6,E12.7,E12.8,E13.2, E13.3,E13.4,E13.5,E13.6,E13.7,E13.8,E14.2,E14.3,E14.4,E14.5, E14.6,E14.7,E14.8
Diabetes Uncomplicated	Diabunc	E10.0,E10.1,E10.9,E11.0,E11.1,E11.9,E12.0,E12.1,E12.9,E13.0, E13.1,E13.9, E14.0,E14.1,E14.9
Drug Abuse	Drug	F11,F12,F13,F14,F15,F16,F18,F19,Z71.5,Z72.2
Fluid & Electrolyte Disorders	FED	E86,E87,E22.2 ,
Hypertension with Chronic Complication	Hypc	I11,I12,I13,I15
Hypertension Uncomplicated	Hypunc	I10
Hypothyroidism	Hypothy	E00,E01,E02,E03,E89.0
Liver Disease	LD	B18,I85,K70,K72,K73,K74,I86.4,I98.2,K71.1,K71.3,K71.4,K71.5,K71.7, K76.0,K76.2, K76.3,K76.4,K76.5,K76.6,K76.7,K76.8,K76.9,Z94.4
Lymphoma	Lymph	C81,C82,C83,C84,C85,C88,C96,C90.0,C90.2
Metastatic Cancer	Metacanc	C77,C78,C79,C80
Obesity	Obes	E66
Other Neurological Disorders	OND	G10,G11,G12,G13,G20,G21,G22,G32,G35,G36,G37,G40,G41,R56, G25.4,G25.5, G31.2,G31.8,G31.9,G93.1,G93.4,R47.0
Paralysis	Para	G81,G82,G04.1,G11.4,G80.1,G80.2,G83.0,G83.1,G83.2,G83.3, G83.4,G83.9,
Peptic Ulcer Dis. exc bleeding	PUD	K25.7,K25.9,K26.7,K26.9,K27.7,K27.9,K28.7,K28.9
Peripheral Vascular Disorders	PVD	I70,I71,I73.1,I73.8,I73.9,I77.1,I79.0,I79.2,K55.1,K55.8,K55.9,Z95.8, Z95.9
Psychoses	Psycho	F20,F22,F23,F24,F25,F28,F29,F30.2,F31.2,F31.5
Pulmonary Circulation Disorders	PCD	I26,I27,I28.0,I28.8,I28.9
Renal Failure	RF	N18,N19,I12.0,I13.1,N25.0,Z49.0,Z49.1,Z49.2,Z94.0,Z99.2
Rheumatoid Arthritis/collagen	Rheumd	M05,M06,M08,M30,M32,M33,M34,M35,M45,L94.0,L94.1,L94.3, M12.0,M12.3, M31.0,M31.1,M31.2,M31.3,M46.1,M46.8,M46.9
Solid Tumor w/o Metastasis	Solidtum	C00,C01,C02,C03,C04,C05,C06,C07,C08,C09,C10,C11,C12,C13, C14,C15,C16,C17, C18,C19,C20,C21,C22,C23,C24,C25,C26,C30, C31,C32,C33,C34, C37,C38,C39,C40, C41,C43,C45,C46,C47, C48, C49,C50,C51,C52,C53,C54,C55,C56, C57,C58,C60,C61, C62,C63, C64,C65,C66,C67,C68,C69,C70,C71,C72,C73,C74,C75, C76,C97
Valvular Disease	Valv	I05,I06,I07,I08,I34,I35,I36,I37,I38,I39,A52.0,I09.1,I09.8,Q23.0, Q23.1, Q23.2,Q23.3,Z95.2,Z95.4
Weight Loss	Wloss	E40,E41,E42,E43,E44,E45,E46,R64,R63.4
