# Supplementary material for: Clustering long-term health conditions among 67728 people with multimorbidity using electronic health records in Scotland
Source: PLoS One. 2023 Nov 29;18(11):e0294666. doi: 10.1371/journal.pone.0294666 (PMC10686427; doi:10.1371/journal.pone.0294666)
Supplement: S2 Table — (PDF) [file pone.0294666.s006.pdf]

S2 Table. Prevalence of the conditions among all patient, patients with multimorbidity and complex multimorbidity

Characteristics	All patients	Multimorbidity (2+)	Complex multimorbidity (4+)
n	318235	67728	20123
AIDS/HIV	0·0	0·1	0·2
Alcohol Abuse	3·8	12·4	16·2
Blood Loss Anaemia	0·1	0·3	0·6
Cardiac Arrhythmia	6·2	24·1	40·8
Chronic Pulmonary Disease	7·6	26·8	39·2
Coagulopathy	0·4	1·5	2·8
Congestive Heart Failure	2·5	10·6	23·1
Deficiency Anaemia	1·7	6·6	13·2
Depression	2·7	10·1	14·6
Diabetes with Chronic Complication	0·5	2·1	5·1
Diabetes Uncomplicated	5·7	23·2	37·5
Drug Abuse	1·3	3·3	3·7
Fluid & Electrolyte Disorders	2·4	10·3	22·2
Hypertension with Chronic Complication	0·1	0·4	1·0
Hypertension Uncomplicated	14·5	53·1	71·4
Hypothyroidism	2·8	10·7	16·5
Liver Disease	1·1	4·4	7·9
Lymphoma	0·7	2·3	3·6
Metastatic Cancer	1·6	6·8	8·9
Obesity	2·0	8·0	14·2
Other Neurological Disorders	2·5	9·1	14·2
Paralysis	0·5	1·9	3·2
Peptic Ulcer Dis· exc bleeding	1·0	3·4	5·5
Peripheral Vascular Disorders	2·0	8·3	16·3
Psychoses	0·8	2·7	3·7
Pulmonary Circulation Disorders	1·2	4·8	9·3
Renal Failure	3·0	13·1	28·8
Rheumatoid Arthritis/collagen	5·0	15·7	21·6
Solid Tumor w/o Metastasis	9·4	27·2	33·2
Valvular Disease	1·9	8·3	17·3
Weight Loss	0·9	3·5	6·4
