# Supplementary material for: Clustering long-term health conditions among 67728 people with multimorbidity using electronic health records in Scotland
Source: PLoS One. 2023 Nov 29;18(11):e0294666. doi: 10.1371/journal.pone.0294666 (PMC10686427; doi:10.1371/journal.pone.0294666)
Supplement: S3 Table — (PDF) [file pone.0294666.s007.pdf]

S3 Table. Prevalence of the conditions by characteristics of people with multimorbidity

Characteristics	All MM	Age					Sex		Deprivation Quintile	
		44-49	50-59	60-69	70-79	80+	F	M	Most	Least
Multimorbidity Patients	67728	3232	10226	14827	19924	19519	36289	31439	13955	12268
**Conditions (Abbreviations)**		%	%	%	%	%	%	%	%	%
AIDS/HIV(AIDS)	0·1	0·4	0·5	0·2	0·0	0·0	0·1	0·2	0·2	0·1
Alcohol Abuse(Alcohol)	12·4	28·0	23·7	15·8	9·2	4·5	9·1	16·2	18·2	7·3
Blood Loss Anemia(Blane)	0·3	0·5	0·5	0·3	0·3	0·3	0·5	0·2	0·4	0·3
Cardiac Arrhythmia(Carit)	24·1	9·6	11·8	16·9	25·7	36·8	20·6	28·1	20·0	27·6
Congestive Heart Failure(CHF)	10·6	3·1	5·2	8·5	11·5	15·3	8·2	13·4	9·8	11·1
Coagulopathy(Coag)	1·5	2·2	1·9	1·6	1·3	1·1	1·4	1·5	1·4	1·5
Chronic Pulmonary Disease(CPD)	26·8	28·8	28·3	28·5	27·3	24·0	28·8	24·6	33·4	21·3
Deficiency Anemia(Dane)	6·6	5·1	5·4	5·3	5·7	9·3	8·3	4·6	7·2	5·8
Depression(Depre)	10·1	28·0	20·4	11·4	6·3	4·6	12·4	7·4	13·1	6·5
Diabetes with Chronic Complication (Diabc)	2·1	2·0	2·6	2·6	1·9	1·7	1·6	2·7	2·5	1·9
Diabetes Uncomplicated(Diabunc)	23·2	14·5	19·5	24·5	25·8	22·9	20·5	26·3	24·9	20·3
Drug Abuse(Drug)	3·3	18·9	8·5	2·8	1·2	0·6	2·7	4·1	5·1	2·4
Fluid & Electrolyte Disorders(FED)	10·3	6·8	6·5	8·4	9·9	14·7	11·5	8·9	10·0	9·9
Hypertension with Chronic Complication (Hypc)	0·4	0·3	0·4	0·4	0·5	0·5	0·4	0·5	0·4	0·5
Hypothyroidism(Hypothy)	10·7	8·2	9·0	10·3	10·5	12·7	16·5	4·1	10·6	10·2
Hypertension Uncomplicated(Hypunc)	53·1	21·0	34·2	49·4	59·5	64·6	52·2	54·1	50·3	55·0
Liver Disease(LD)	4·4	7·0	6·9	5·6	3·8	2·2	4·4	4·3	5·0	3·6
Lymphoma(Lymph)	2·3	1·7	1·7	2·4	2·8	2·0	1·9	2·6	2·0	2·5
Metastatic Cancer(Metacanc)	6·8	4·8	6·8	7·6	7·1	6·2	8·1	5·3	5·2	8·3
Obesity(Obes)	8·0	11·4	12·2	10·6	7·7	3·6	8·3	7·6	10·0	5·7
Other Neurological Disorders(OND)	9·1	15·8	12·2	9·2	7·6	7·6	8·7	9·5	10·0	7·7
Paralysis(Para)	1·9	2·6	2·7	2·1	1·6	1·5	1·9	1·9	2·0	1·7
Pulmonary Circulation Disorders(PCD)	4·8	4·1	4·1	4·1	4·9	5·6	4·8	4·7	4·9	4·9
Psychoses(Psycho)	2·7	9·1	5·6	2·9	1·6	1·1	2·6	2·8	3·4	1·7
Peptic Ulcer Dis· exc bleeding(PUD)	3·4	2·5	3·2	3·4	3·6	3·4	3·0	3·9	3·9	2·8
Peripheral Vascular Disorders(PVD)	8·3	2·7	4·8	7·5	9·7	10·3	6·2	10·7	9·4	7·0
Renal Failure(RF)	13·1	4·7	4·8	6·9	12·4	24·3	14·5	11·5	12·1	13·5
Rheumatoid Arthritis/collagen(Rheumd)	15·7	10·5	13·1	15·2	17·3	16·5	15·4	15·9	14·3	17·1
Solid Tumor w/o Metastasis(Solidtum)	27·2	12·1	16·8	24·4	31·0	33·5	28·7	25·6	22·6	32·7
Valvular Disease(Valv)	8·3	3·1	4·2	5·6	8·7	13·0	7·5	9·2	6·9	9·8
Weight Loss(Wloss)	3·5	3·8	3·3	3·2	3·2	4·2	3·9	3·1	4·3	2·8
