# Supplementary material for: Clustering long-term health conditions among 67728 people with multimorbidity using electronic health records in Scotland
Source: PLoS One. 2023 Nov 29;18(11):e0294666. doi: 10.1371/journal.pone.0294666 (PMC10686427; doi:10.1371/journal.pone.0294666)
Supplement: S4 Table — (PDF) [file pone.0294666.s008.pdf]

S4 Table. Multimorbidity Clusters of the Conditions among the whole multimorbid population, sex and deprivation subgroups

Population subgroup	Cluster	Conditions*	No of people in cluster n(%)	Mean age (std dev)	Women %	% in Most Deprived	% in Least Deprived
**Whole Population** N=67728	1	Alcohol Abuse<br>Other Neurological Disorders<br>Depression	17366 (25·6)	66·4 (12·5)	8891 (51·2)	4098 (23·6)	2049 (11·8)
	2	Solid Tumour w/o Metastasis<br>Metastatic Cancer	19123 (28·2)	74·7 (11·1)	10766 (56·3)	2945 (15·4)	3748 (19·6)
	3	Obesity<br>Chronic Pulmonary Disease<br>Uncomplicated Hypertension<br>Uncomplicated Diabetes<br>Rheumatoid Arthritis/Collagen<br>Hypothyroidism	55105 (81·4)	72·6 (12)	29757 (54)	10250 (18·6)	8872 (16·1)
	4	Peripheral Vascular Disorders<br>Renal Failure<br>Fluid & Electrolyte Disorders<br>Deficiency Anaemia	20771 (30·7)	75·8 (11·9)	11736 (56·5)	3884 (18·7)	3199 (15·4)
	5	Valvular Disease<br>Congestive Heart Failure<br>Cardiac Arrhythmia Pulmonary<br>Circulation Disorders	23497 (34·7)	75·7 (12)	11020 (46·9)	3854 (16·4)	4229 (18·0)
**Men** Population N=31439	1	Alcohol abuse<br>Other Neurological Disorders<br>Depression<br>Liver Disease	8470 (26·9)	66·6 (12)	na	2067 (24·4)	974 (11·5)
	2	Solid Tumour w/o Metastasis<br>Metastatic Cancer	8364 (26·6)	75·3 (10·3)	na	1230 (14·7)	1723 (20·6)
	3	Obesity<br>Chronic Pulmonary Disease<br>Uncomplicated Hypertension<br>Uncomplicated Diabetes<br>Rheumatoid Arthritis/Collagen	25085 (79·8)	72·1 (11·2)	na	4565 (18·2)	4239 (16·9)
	4	Peripheral Vascular Disorders<br>Renal Failure<br>Fluid & Electrolyte Disorders	8202 (26·1)	74·9 (11·1)	na	1517 (18·5)	1312 (16)
	5	Valvular Disease<br>Congestive Heart Failure<br>Cardiac Arrhythmia	11693 (37·2)	75 (10·8)	na	1812 (15·5)	2233 (19·1)
**Women** Population N=36289	1	Alcohol Abuse<br>Other Neurological Disorders<br>Depression	8896 (24·5)	66·3 (13)	na	2028 (22·8)	1068 (12)
	2	Solid Tumour w/o Metastasis<br>Metastatic Cancer	10759 (29·6)	74·3 (11·7)	na	1711 (15·9)	2012 (18·7)
	3	Obesity<br>Chronic Pulmonary Disease<br>Uncomplicated Hypertension<br>Uncomplicated Diabetes<br>Rheumatoid Arthritis/Collagen<br>Hypothyroidism	29750 (82)	73 (11·5)	na	5623 (18·9)	11722 (39·4)
	4	Peripheral Vascular Disorders<br>Renal Failure<br>Fluid & Electrolyte Disorders<br>Deficiency Anaemia	11735 (32·3)	76·6 (12·4)	na	2183 (18·6)	1772 (15·1)
	5	Valvular Disease<br>Congestive Heart Failure<br>Cardiac Arrhythmia<br>Pulmonary Circulation Disorders	11012 (30·3)	76·9 (12)	na	1872 (17)	1850 (16·8)
**Most Deprived** Population N=13955	1	Alcohol abuse<br>Other Neurological Disorders<br>Depression<br>Liver Disease<br>Drug Abuse	4631 (33·2)	63·5 (11·7)	2302 (49·7)	na	na

	2	Solid Tumour w/o Metastasis<br>Metastatic Cancer	2943<br>(21·1)	73·2<br>(11·5)	1713<br>(58·2)	na	na
	3	Obesity<br>Chronic Pulmonary Disease<br>Uncomplicated Hypertension<br>Uncomplicated Diabetes	9523<br>(68·2)	70·2<br>(12)	5181<br>(54·4)	na	na
	4	Peripheral Vascular Disorders<br>Renal Failure<br>Fluid & Electrolyte Disorders<br>Deficiency Anaemia<br>Hypothyroidism<br>Rheumatoid Arthritis/Collagen	3850<br>(27·6)	73·2<br>(11·9)	1875<br>(48·7)	na	na
	5	Valvular Disease<br>Congestive Heart Failure Cardiac Arrhythmia<br>Pulmonary Circulation Disorders	5912<br>(42·4)	71·8<br>(12·3)	3494<br>(59·1)	na	na
**Least** Deprived Population N=12268	1	Solid Tumour w/o Metastasis<br>Metastatic Cancer	2044<br>(16·7)	70·1<br>(12·5)	1069<br>(52·3)	na	na
	2	Obesity<br>Chronic Pulmonary Disease<br>Cardiac Arrhythmia<br>Uncomplicated Hypertension<br>Uncomplicated Diabetes<br>Rheumatoid Arthritis/Collagen<br>Hypothyroidism	3739<br>(30·5)	75·7<br>(10·7)	2015<br>(53·9)	na	na
	3	Peripheral Vascular Disorders<br>Renal Failure<br>Fluid & Electrolyte Disorders<br>Deficiency Anaemia	9539<br>(77·8)	74·9<br>(11·3)	4846<br>(50·8)	na	na
	4	Valvular Disease<br>Congestive Heart Failure<br>Pulmonary Circulation Disorders	3209<br>(26·2)	78·1<br>(11·2)	1775<br>(55·3)	na	na
	5	Solid Tumour w/o Metastasis<br>Metastatic Cancer	2300<br>(18·7)	77·4<br>(10·7)	1010<br>(43·9)	na	na

*only Conditions with at least 5% prevalence within the specific population subgroup were clustered na not applicable
