# Supplementary material for: Clustering long-term health conditions among 67728 people with multimorbidity using electronic health records in Scotland
Source: PLoS One. 2023 Nov 29;18(11):e0294666. doi: 10.1371/journal.pone.0294666 (PMC10686427; doi:10.1371/journal.pone.0294666)
Supplement: S5 Table — (PDF) [file pone.0294666.s009.pdf]

S5 Table. Multimorbidity Clusters of the Conditions across age subgroups

Population subgroup	Cluster	Conditions*	No of people in cluster, n,%	Mean age (std dev)	Women %	% in Most Deprived	% in Least Deprived
**Age 44-49 Years** N=3232	1	Alcohol abuse<br>Other Neurological Disorders<br>Depression<br>Psychoses<br>Drug Abuse<br>Liver Disease	2193 (67·9)	47·1(1·4)	1226 (55·9)	658 (30)	184 (8·4)
	2	Solid Tumour w/o Metastasis<br>Fluid & Electrolyte Disorders<br>Cardiac Arrhythmia<br>Rheumatoid Arthritis/Collagen<br>Deficiency Anaemia<br>Hypothyroidism	1409 (43·6)	47·2 (47·2)	881 (62·5)	373 (26·5)	159 (11·3)
	3	Obesity<br>Uncomplicated Hypertension<br>Uncomplicated Diabetes	1005 (31·1)	47·1(1·4)	573 (57)	266 (26·5)	91 (9·1)
**50-59 Years** N=10226	1	Alcohol abuse<br>Depression<br>Psychoses<br>Drug Abuse	4230 (42·2)	54·7(2·9)	2233 (52·8)	1252 (29·6)	330 (7·8)
	2	Solid Tumour w/o Metastasis<br>Metastatic Cancer	1679 (16·7)	55·2(2·4)	1137 (67·7)	321 (19·1)	264 (15·7)
	3	Obesity<br>Chronic Pulmonary Disease<br>Uncomplicated Hypertension<br>Uncomplicated Diabetes<br>Rheumatoid Arthritis/Collagen<br>Hypothyroidism	7122 (71)	55·1(2·8)	4038 (56·7)	1788 (25·1)	791 (11·1)
	4	Fluid & Electrolyte Disorders<br>Deficiency Anaemia<br>Liver Disease<br>Other Neurological Disorders	2737 (27·3)	54·9(2·8)	1560 (57)	747 (27·3)	268 (9·8)
	5	Valvular Disease<br>Congestive Heart Failure<br>Cardiac Arrhythmia	1497 (14·9)	55·4(2·8)	651 (43·5)	323 (21·6)	195 (13)
**60-69 Years** N=14827	1	Alcohol abuse<br>Other Neurological Disorders<br>Depression<br>Liver Disease	4846 (32·7)	64·5(2·9)	2438 (50·3)	1163 (24)	548 (11·3)
	2	Solid Tumour w/o Metastasis<br>Metastatic Cancer	3656 (24·7)	65(2·9)	2099 (57·4)	632 (17·3)	629 (17·2)
	3	Obesity<br>Chronic Pulmonary Disease<br>Uncomplicated Hypertension<br>Uncomplicated Diabetes<br>Rheumatoid Arthritis/Collagen<br>Hypothyroidism	11761 (79·3)	64·4(2·9)	5963 (50·7)	2493 (21·2)	1623 (13·8)
	4	Peripheral Vascular Disorders<br>Renal Failure<br>Fluid & Electrolyte Disorders<br>Deficiency Anaemia	3408 (23)	64·9(2·8)	1728 (50·7)	770 (22·6)	440 (12·9)
	5	Valvular Disease<br>Congestive Heart Failure Cardiac Arrhythmia	3468 (43·4)	65(2·8)	1318 (38)	652 (18·8)	534 (15·4)
**70-79 Years** N=19924	1	Alcohol abuse<br>Other Neurological Disorders<br>Depression	4046 (20·3)	74·1(2·8)	1845 (45·6)	825 (20·4)	570 (14·1)
	2	Solid Tumour w/o Metastasis<br>Metastatic Cancer	6269 (31·5)	74·5(2·8)	3279 (52·3)	947 (15·1)	1279 (20·4)
	3	Chronic Pulmonary Disease<br>Uncomplicated Hypertension<br>Uncomplicated Diabetes<br>Rheumatoid Arthritis/Collagen	15069 (75·6)	74·5(2·8)	7218 (47·9)	2592 (17·2)	2682 (17·8)

	4	Peripheral Vascular Disorders<br>Renal Failure<br>Fluid & Electrolyte Disorders<br>Deficiency Anaemia	5972 (30)	74·6(2·8)	3004 (50·3)	1147 (19·2)	896 (15)
	5	Valvular Disease<br>Congestive Heart Failure<br>Cardiac Arrhythmia	6719 (33·7)	74·7(2·8)	2674 (39·8)	1122 (16·7)	1250 (18·6)
	6	Obesity<br>Hypothyroidism Pulmonary<br>Circulation Disorders	4309 (21·6)	74·3(2·8)	2749 (63·8)	814 (18·9)	702 (16·3)
**80+ Years**<br>N=19519	1	Solid Tumour w/o Metastasis<br>Uncomplicated Hypertension<br>Uncomplicated Diabetes<br>Chronic Pulmonary Disease<br>Cardiac Arrhythmia<br>Renal Failure	19395 (99·4)	86(4·6)	11307 (58·3)	2735 (14·1)	3743 (19·3)
	2	Peripheral Vascular Disorders<br>Pulmonary Circulation Disorders<br>Congestive Heart Failure<br>Valvular Disease	6770 (34·7)	86(4·6)	3581 (52·9)	948 (14)	1307 (19·3)
	3	Metastatic Cancer<br>Other Neurological Disorders<br>Fluid & Electrolyte Disorders<br>Hypothyroidism<br>Deficiency Anaemia<br>Rheumatoid Arthritis/Collagen	10425 (53·4)	86·1(4·7)	6578 (63·1)	1501 (14·4)	1991 (19·1)

*only Conditions with at least 5% prevalence within the specific population subgroup were clustered
