# Supplementary material for: Clustering long-term health conditions among 67728 people with multimorbidity using electronic health records in Scotland
Source: PLoS One. 2023 Nov 29;18(11):e0294666. doi: 10.1371/journal.pone.0294666 (PMC10686427; doi:10.1371/journal.pone.0294666)
Supplement: S6 Table — (PDF) [file pone.0294666.s010.pdf]

S6 Table. Multimorbidity Clusters of the Conditions across age-deprivation subgroups

Population subgroup	Cluster	Conditions*	No of people in cluster: n%	Mean age (std dev)	Women n%
**Age 44-49 Years& Most Deprived** N=811	1	Alcohol Abuse<br>Drug Abuse<br>Depression<br>Liver Disease<br>Other Neurological Disorders<br>Psychoses<br>Chronic Pulmonary Disease	670 (82·6)	47·1(1·5)	366 (54·6)
	2	Obesity<br>Uncomplicated Hypertension<br>Uncomplicated Diabetes	266 (32·8)	47·2(1·5)	161 (60·5)
	3	Solid Tumour W/o Metastasis<br>Fluid & Electrolyte Disorders<br>Cardiac Arrhythmia<br>Hypothyroidism Rheumatoid Arthritis/collagen	290 (35·8)	47·1(1·5)	161 (55·5)
**44-49 Years & Least Deprived** N=280	1	Alcohol abuse<br>Drug Abuse<br>Depression<br>Psychoses	117 (41·8)	47·2(1·4)	66 (56·4)
	2	Solid Tumour W/o Metastasis<br>Metastatic Cancer	63 (22·5)	47·2(1·5)	45 (71·4)
	3	Obesity<br>Uncomplicated Hypertension<br>Uncomplicated Diabetes<br>Other Neurological Disorders<br>Hypothyroidism<br>Chronic Pulmonary Disease	171 (61·1)	47·2(1·4)	96 (56·1)
	4	Cardiac Arrhythmia<br>Deficiency Anaemia<br>Hypothyroidism	77 (27·5)	47·1(1·5)	44 (57·1)
	5	Liver Disease<br>Renal Failure<br>Fluid & Electrolyte Disorders	44 (15·7)	47·1(1·4)	19 (43·2)
**80+ Years & Most Deprived** N=2839	1	Solid Tumour W/o Metastasis<br>Metastatic Cancer	965 (34)	85·7(4·6)	584 (60·5)
	2	Alcohol abuse<br>Drug Abuse<br>Depression<br>Psychoses	581 (20·5)	85·2(4·3)	316 (54·4)
	3	Obesity<br>Uncomplicated Hypertension<br>Uncomplicated Diabetes<br>Other Neurological Disorders<br>Hypothyroidism<br>Chronic Pulmonary Disease	1653 (58·2)	85·3(4·4)	1015 (61·4)
	4	Cardiac Arrhythmia<br>Deficiency Anaemia<br>Rheumatoid Arthritis/collagen	1462 (51·5)	85·8(4·5)	835 (57·1)
	5	Liver Disease<br>Renal Failure<br>Fluid & Electrolyte Disorders	1025 (36·1)	86·3(4·8)	687 (67)
**80+ Years & Least Deprived** Population N=3933	1	Solid Tumour W/o Metastasis<br>Uncomplicated Hypertension<br>Uncomplicated Diabetes<br>Chronic Pulmonary Disease<br>Fluid & Electrolyte Disorders<br>Cardiac Arrhythmia<br>Renal Failure<br>Rheumatoid Arthritis/collagen	3826 (97·3)	86(4·6)	2127 (55·6)
	2	Pulmonary Circulation Disorders<br>Congestive Heart Failure<br>Valvular Disease	1084 (27·6)	86·3(4·7)	556 (51·3)

	3	Metastatic Cancer<br>Other Neurological Disorders<br>Peripheral Vascular Disorders<br>Hypothyroidism<br>Deficiency Anaemia	1422 (36·2)	86·1(4·6)	894 (62·9)

*only Conditions with at least 5% prevalence within the specific population subgroup were clustered
