# Supplementary material for: Clustering long-term health conditions among 67728 people with multimorbidity using electronic health records in Scotland
Source: PLoS One. 2023 Nov 29;18(11):e0294666. doi: 10.1371/journal.pone.0294666 (PMC10686427; doi:10.1371/journal.pone.0294666)
Supplement: Attachment — Submitted filename: MM in Scotland_Clusters.docx [file pone.0294666.s011.docx]

**Clustering long-term health conditions among 67728 people with multimorbidity using electronic health records in Scotland**

This paper used 20 years of linked electronic health records to examine clusters of multimorbidity in Scotland across age groups, sex, and levels of deprivation. Looking at how conditions cluster together across different strata in society will potentially have implications for health service delivery. This paper contains important work but presents many complex findings which can make it difficult to interpret at times. Therefore, this paper requires restructuring and some considerable changes before it can be considered for publication.

***Abstract***

You say multimorbidity is present in 21.3% and then later on you say ‘multimorbidity was present in one of every 5 people’. This is repetition. Consider removing one sentence.

Please reconsider how you describe the multimorbidity clusters in the Abstract as it is confusing in its current form. I would recommend naming the five distinct clusters and then mention that they differ by age, sex, and deprivation without going into too much detail about this in the Abstract.

There are more incidences of repetition in the Abstract – please address this.

***Background***

The Background is well-structured and you build your argument well.

Please provide a definition of ‘complex multimorbidity’ in order to justify the aims of your study.

Further justification is required for using data from Scotland. For example, the strong socioeconomic gradient in Scotland might be one reason for focusing on this country (example in <https://www.nature.com/articles/s41598-022-20546-4#ref-CR18>)

***Methods***

Please clarify – if participants comprised all those who were alive on 31^st^ December 2018, how did you use death records to measure multimorbidity?

It would be helpful to provide justification for the use of the Elixhauser Comorbidity Index to measure multimorbidity when there are more comprehensive measures available now, e.g. <https://bmjmedicine.bmj.com/content/bmjmed/1/1/e000247.full.pdf>

In the Outcome section, please provide some examples of physical and mental long-term conditions that were included in the multimorbidity definition.

Line 119 – frequencies and proportions of what? Numbers of conditions? This needs to be more clear.

The role of complex multimorbidity in this study is not clear from the Methods- particularly the Data Management and Statistical Analysis section. Please provide more clarity.

Could you include a sentence or two in the Data Management and Statistical Analysis section about Market Basket Analysis (MBA) i.e. how this clustering method allows for clustering across the characteristics of the study sample? I believe hierarchical cluster analysis can do the same thing so it would be good to further justify the use of MBA.

***Results***

In the Methods you state that the study was based on ‘cross-sectional analysis…when an individual was aged at least 25 years’ (line 90/91). However, the sample in the Results comprise people aged over 44 years. Some clarity is required here. Why over 44 years? Is this because there is a retrospective study period of 20 years?

Were those with complex multimorbidity included in the overall multimorbidity count? i.e. Did 67,728 people have MM or did 87,851 people have MM? Please provide clarity in the paper.

Table 1 – please provide proportions (%) for ‘All individuals’, e.g., for sex, age groups, etc. to allow comparison with the MM group. Please also provide a mean age for ‘All individuals’.

Figure 1 – I would recommend removing the percentages attached to the lines in the graph

In the Introduction, you talk about links between MM and obesity and harmful use of alcohol and drugs (lines 58-60). However, you include both obesity and alcohol abuse as long-term conditions in your multimorbidity measure. I would consider removing this from the Introduction as it introduces confusion.

It might be useful to assign more meaningful names to the clusters, seeing as they don’t differ massively between sex or deprivation levels. For example, cluster 5 could be called the cardiovascular cluster.

Lines 181 to 190 are confusing for the reader. Please consider presenting these results in a different way.

Line 191-192: Please provide some examples of the core set of conditions across strata

Table 2 is really helpful for the interpretation of Results. However, I would suggest reorganising the ‘Clustering’ section of the Results for more clarity. More subheadings would be useful. I would consider basing these on individual clusters.

Why was there no cluster analysis performed amongst those with complex multimorbidity?

Presenting clusters across age groups is difficult and I think Table 3 requires some further clarification. Perhaps include a footnote to indicate that efforts were made to align clusters that were similar. I would also explain why blank cells exist for certain age groups. Moreover, would there be a way of showing how common it is to for participants with MM to belong to one of these clusters in the Table?

***Discussion***

In the second paragraph, you state that 99% of those aged >80 years were included in cluster 1- please correct to Cluster 2. And correct ‘clusters 2 and 3’ to ‘4 and 6’ (judging by Table 3).

Parts of the Discussion feel like a Results section (but for the Supplementary results). Make sure to discuss results here, rather than just describe them.

Please add some strengths of the study

Are there any limitations relating to market basket analysis that need to be mentioned? Is this widely applied to healthcare data?

Are there any limitations around the measure of deprivation used?

***General remarks:***

Please check spelling and grammar throughout
