# Supplementary material for: Clustering long-term health conditions among 67728 people with multimorbidity using electronic health records in Scotland
Source: PLoS One. 2023 Nov 29;18(11):e0294666. doi: 10.1371/journal.pone.0294666 (PMC10686427; doi:10.1371/journal.pone.0294666)
Supplement: Attachment — Submitted filename: Comments to the Author R2.docx [file pone.0294666.s013.docx]

**Comments to the Author**

Reviewer #2: (No Response)

Reviewer #3: Title: Clustering long-term health conditions among 67728 people with multimorbidity using electronic health records in Scotland
Manuscript ID: PONE-D-23-03161R

The authors have clearly put in quite a bit of effort to address the Reviewer’s concerns. I appreciate that they have clarified their methods and presentation of their results. There are still a few issues that I would like to see addressed:

1. It is not clear to me why Cluster 1 is characterized as Alcohol Abuse. I would like to see some rationale for this decision. For the other clusters, the reasoning for the label is more clear or seems to be more representative of the main conditions but less so for Cluster 1. I think because a label like alcohol abuse could be considered stigmatizing, and the way it is presented with economic deprivation, makes me somewhat uncomfortable. I also wonder if there is something to be discussed in terms of how codes such as alcohol abuse are assigned and whether that is at all related to the observed patterns with age and economic deprivation.

The naming of the clusters was discussed with the research team with either the most common condition or a representative term used but it is still subjective labelling.  Cluster 1 for instance was named as alcohol misuse as this was the most common condition for people identified in the cluster but it could also have been labelled as socioeconomic-driven conditions
2. I think that the Discussion would benefit from some more thorough editing. There are some places where the sentences are not complete – I think this is likely from the multiple rounds of track changes – but it does make it difficult to follow even the clean version. It is also repetitive in places, particularly the points about clustering showing variability by age and economic deprivation.

We have edited the discussion and removed duplications
3. Related to the above, I think that the authors can provide a deeper discussion of what the clusters might say about why they may be seeing these conditions together – either because they are physiologically related, have common determinants, or some other reason. I do not think as currently presented there is much offered beyond describing the common conditions in clusters.

We have edited the section as well. Our analysis shows association not causality, but it may be possible to surmise the drivers of specific clusters as identified.  Conditions most prevalent in our most deprived population groups include alcohol and drug misuse, depression and obesity which are all know to be associated with social factors.  Other identified clusters are likely to have more physiological drivers e.g. hypertension through to heart diseases.
4. I do not understand the statement that Market Basket Analysis is based on patterns in the data but may not guarantee associations (page 15, line 372).

Thank you, We have changed this line to now read “However, the clusters generated by market basket analysis are based on empirical patterns in the data and only show associations between different conditions, it does not show any causal relationships between those data.”
